# Gender Stereotypes in a Children's Television Program: Effects on Girls' and Boys' Stereotype Endorsement, Math Performance, Motivational Dispositions, and Attitudes

**DOI:** 10.3389/fpsyg.2018.02435

**Published:** 2018-12-04

**Authors:** Eike Wille, Hanna Gaspard, Ulrich Trautwein, Kerstin Oschatz, Katharina Scheiter, Benjamin Nagengast

**Affiliations:** ^1^Hector Research Institute of Education Sciences and Psychology, University of Tübingen, Tübingen, Germany; ^2^Leibniz-Institut für Wissensmedien, Tübingen, Germany; ^3^University of Tübingen, Tübingen, Germany

**Keywords:** stereotypes, gender differences, television, math motivation, math performance

## Abstract

Television programs are a central part of children's everyday lives. These programs often transmit stereotypes about gender roles such as “math is for boys and not for girls.” So far, however, it is unclear whether stereotypes that are embedded in television programs affect girls' and boys' performance, motivational dispositions, or attitudes. On the basis of research on expectancy-value theory and stereotype threat, we conducted a randomized study with a total of 335 fifth-grade students to address this question. As the experimental material, we used a television program that had originally been produced for a national TV channel. The program was designed to show children that math could be interesting and fun. In the experimental condition, the program included a gender stereotyped segment in which two girls who were frustrated with math copied their math homework from a male classmate. In the control condition, participants watched an equally long, neutral summary of the first part of the video. We investigated effects on boys' and girls' stereotype endorsement, math performance, and different motivational constructs to gain insights into differential effects. On the basis of prior research, we expected negative effects of watching the stereotypes on girls' performance, motivational dispositions, and attitudes. Effects on the same outcomes for boys as well as children's stereotype endorsement were explored as open questions. We pre-registered our research predictions and analyses before conducting the experiment. Our results provide partial support for short-term effects of gender stereotypes embedded in television programs: Watching the stereotypes embedded in the video increased boys' and girls' stereotype endorsement. Boys reported a higher sense of belonging but lower utility value after watching the video with the stereotypes. Boys' other outcome variables were not affected, and there were also no effects on girl's performance, motivational dispositions, or attitudes. Results offer initial insights into how even short segments involving gender stereotypes in television shows can influence girls' and boys' stereotype endorsement and how such stereotypes may constitute one factor that contributes to gender differences in the STEM fields.

## Introduction

Women are underrepresented in domains that require intensive mathematical skills (National Science Foundation, 2015; National Science Board, 2016). This bias is crucial to the larger economy and contributes to gender inequity in income: More women in science, technology, engineering, and mathematics (STEM) would diversify the workforce, and mathematically intensive STEM fields usually provide high-status career options (National Science Foundation, 2015). Drawing on expectancy-value theory (Eccles et al., 1983), gender differences in STEM careers can be linked to early emerging gender differences in math motivational dispositions. These are rooted in different socialization processes for girls and boys such as the gender stereotypes children encounter in their environments (see Wigfield et al., 2015). Research on stereotype threat has provided insights into the potential mechanisms behind how gender stereotypes might affect girls and boys, indicating that girls can show lower math performance and motivation in the short-term if they are reminded of the stereotype that females perform worse than males in math, whereas boys' performance can benefit from such stereotypes (for a review, see Spencer et al., 2016).

Television programs are one potential source of gender stereotypes for children. Despite the wide diversity of media available nowadays, television continues to be one of the most popular and widely used media among children (Rideout, 2015; Feierabend et al., 2017). Television shows and programs with STEM content have increased in availability (National Reserach Council., 2009) and popularity (Patten, 2013) within the last decade. They transmit certain beliefs and stereotypes about gender roles in the STEM field, such as showing females as underperforming in math and science (Collins, 2011). It is not yet clear, though, whether stereotypes in television programs affect girls' and boys' performance and motivational dispositions in math. So far, research on expectancy-value theory has focused primarily on the role of stereotypes that are implicitly conveyed by parents, teachers, or peers (see Wigfield et al., 2015), whereas research on stereotype threat has traditionally investigated effects of stereotypes presented as isolated stimuli in laboratory settings with a primary focus on adult samples (see Spencer et al., 2016).

In the present study, we aimed to contribute to closing this gap in the literature by examining effects of traditional gender stereotypes in a math television program for children. To increase the ecological validity of the study, we used a television program that was broadcast on a German national TV channel. Specifically, the end of this program showed two girls who were not doing well in math and copied their homework from a male classmate. To examine the effects of these stereotypes, we conducted a randomized study with a pretest–posttest design in which fifth graders watched this television program about math either with or without the segment in which these gender stereotypes were portrayed. In order to comprehensively investigate possible effects, we studied effects on both girls' and boys' stereotype endorsement as well as their performance, motivational dispositions (i.e., expectancy and value beliefs), and attitudes toward math (i.e., sense of belonging, feelings about the domain).

### Gender Differences in Motivational Dispositions and Achievement in Math From an Expectancy-Value Theory Perspective

#### Expectancy-Value Theory

Eccles et al. (1983) expectancy-value theory is one of the most widely used frameworks for investigating gender differences in motivational dispositions in math and has been highly effective in explaining women's underrepresentation in the STEM fields (Watt and Eccles, 2008; Schoon and Eccles, 2014).

In general, motivation can be defined as “the process whereby goal-directed activity is instigated and sustained” (Schunk et al., 2008, p.4). However, current work on motivation from the perspective of expectancy-value theory focusses mainly on expectancy and value beliefs as motivational dispositions (Eccles et al., 1983; Eccles, 2005). Specifically, Eccles et al. (1983) suggested that the expectation of success in a specific domain as well as several aspects of subjective task values would predict academic decision making and thereby also specific educational outcomes, such as later achievement or educational choices. Young people should thus choose math-intensive STEM careers if they expect to be good at math and science activities and have high values in these domains.

Eccles and Wigfield (2002) defined expectancies for success as a person's beliefs about his or her success in a task in the immediate or long-term future. Expectancy beliefs are therefore closely related to other competence beliefs, such as academic self-concept, which has often been used to measure expectancies for success (see Marsh, 2007; Nagengast et al., 2011). Eccles et al. (1983) differentiated four different components of subjective task values: intrinsic value, attainment value, utility value, and cost. Intrinsic value is defined as enjoyment while performing a task (Eccles, 2005). It is thus similar to other motivational constructs such as intrinsic motivation as defined by Deci and Ryan (1985)—which refers to reasons for engaging in a task, such as inherent satisfaction—or interest as defined by Renninger and Hidi (2011). Attainment value refers to the personal importance of doing well on a task or in a domain (Eccles, 2005). Utility value captures more extrinsic reasons for engaging in a task, namely the perceived usefulness of a task or domain (Eccles, 2005). Finally, cost captures negative aspects of engaging in a task or domain, such as required effort or time (Eccles, 2005).

#### Gender Differences in Motivational Dispositions and Achievement in Math

Ample research drawing upon expectancy-value theory has consistently indicated that girls exhibit lower expectancy and value beliefs (and higher cost) for math than boys from an early age on (for reviews, see Wang and Degol, 2013; Wigfield et al., 2015). By contrast, meta-analyses investigating gender differences in math achievement have shown rather small advantages for boys compared with girls (e.g., Else-Quest et al., 2010; Reilly et al., 2015). Moreover, these analyses have indicated that such gender differences seem to occur only on math achievement tests (Reilly et al., 2015), whereas girls even show an advantage in teacher-assigned school marks (Voyer and Voyer, 2014).

### The Role of Stereotypes in the Development of Children's Motivational Dispositions and Achievement

According to expectancy-value theory, socializers' beliefs and behaviors as well as cultural milieu influence individuals' task perceptions and interpretations of previous academic achievement (Eccles et al., 1983). In explaining gender differences in expectancy and value beliefs and achievement, expectancy-value theory thus indicates that girls and boys are socialized through different processes, which are shaped by the surrounding environment and its gender norms and roles, the individuals' beliefs, and the choices females and males make on the basis of their socialization (Eccles, 2009). In particular, gendered socialization refers to specific gender roles or the gender-stereotypical attitudes and expectancies of parents, teachers, and other socializing influences such as the media, all of which transmit gender stereotypes (Wigfield et al., 2015).

Stereotypes can be broadly defined as associations of group members with specific attributes (Greenwald et al., 2002). Regarding gender, there are specific stereotypes about the traits, abilities, and motivation of males and females, specifically in the domain of math (see Leaper, 2015). Math and science are male-typed domains, and gender stereotypes in these domains include assumptions about lower abilities and less talent in math for females compared with males (e.g., Spencer et al., 1999).

According to expectancy-value theory, as a result of the gender stereotypes children face in their socialization, girls disidentify with math and devalue the subject in the long run, whereas boys may particularly identify with and value math (Eccles et al., 1983; Wigfield et al., 2015). Consequently, boys develop higher competence beliefs and values in male-typed domains such as math and math-intensive STEM domains, whereas girls develop higher competence beliefs and values in female-typed domains such as languages and arts (e.g., Wigfield et al., 2015). It is assumed that such gender differences in math competence beliefs and values may lead to gender differences in math achievement in the long run (Wigfield and Eccles, 2000). Previous studies have supported these assumptions by showing that women's gender stereotypes reduced their domain identification (e.g. their positive attitudes and their sense of belonging; Cheryan et al., 2009; see also Thoman et al., 2013 for a review) as well as their future expectancies of success (Smith et al., 2015) and their future task values (Plante et al., 2013; Smith et al., 2015). Expectancy and task values, in turn, have been shown to be important predictors of later achievement (e.g., Marsh et al., 2005; Denissen et al., 2007).

### Stereotype Threat as a Potential Mechanism for How Stereotypes can Influence Children

The repeated experience of stereotypes is one potential mechanism that may explain how stereotypes of others can influence girls' and boys' performance, expectancy and value beliefs, and attitudes toward math. According to expectancy-value theory, such experiences might lead to the internalization of gender-role stereotypes, with the previously described consequences that girls disidentify with and devalue math, and boys particularly identify with and value math in the long run (Eccles et al., 1983; Wigfield et al., 2015).

Research on stereotype threat has provided support for this idea by showing that the activation of traditional gender stereotypes can reduce girls' attitudes and belonging in math as well as their performance and motivational dispositions in the short term (for a review, see Spencer et al., 2016). Steele and Aronson (1995) defined stereotype threat as a situational experience in which group members feel concerned about confirming a negative stereotype that pertained to their own group. They suggested that such concerns might compromise a person's behavior and performance.

#### Stereotype Threat and Girls' Performance, Motivational Dispositions, and Attitudes

Originally, research on stereotype threat focused on explaining the underperformance of African Americans in performance (Steele and Aronson, 1995), but ample research has also been conducted to examine gender differences in math-intensive domains (e.g., Spencer et al., 1999; Schmader, 2002; Tomasetto et al., 2011). Such research has demonstrated that females show lower math performance if they are reminded of negative stereotypes about women in math, but they perform as well as males if such stereotypes are not made salient before they take a math test (Nguyen and Ryan, 2008; Doyle and Voyer, 2016). Although most of this research has been conducted on college students or older adults, multiple studies have reported similar effects among children or adolescents (e.g., Ambady et al., 2001; Flore and Wicherts, 2015). These studies have demonstrated that children in elementary school are already aware of their own gender and show gender-stereotypical views in the domain of math, as they attribute lower math ability and talent to girls and women than to boys and men (e.g., Signorella et al., 1993; Ambady et al., 2001; Passolunghi et al., 2014). In addition, there is research on the short-term effects of stereotypes on math performance among girls of different ages (Ambady et al., 2001; Muzzatti and Agnoli, 2007; Neuville and Croizet, 2007; Tomasetto et al., 2011; Hermann and Vollmeyer, 2016). A meta-analysis by Flore and Wicherts (2015), for instance, found that girls who are reminded of typical gender stereotypes in math exhibit slightly lower math performance compared to girls who are not reminded of such stereotypes. Such effects have been consistently found for girls younger than 13 years old.

Effects of stereotype threat have also been shown for females' motivational dispositions and attitudes toward a domain, such as their domain identification and their sense of belonging in math and science (e.g., Cheryan et al., 2009; see also Thoman et al., 2013, for a review), their competence beliefs (Cadinu et al., 2003), and their interest (Smith et al., 2007; see also Thoman et al., 2013, for a review). Again, much of this work has been conducted on adult samples. However, there are a few studies reporting similar effects for girls. A study by Muzzatti and Agnoli (2007) indicated stereotype threat effects on 8th grade girls' competence beliefs in math, although no effects were found for 3rd and 5th graders. Furthermore, Master et al. (2015) found stereotype threat effects on 15-years-old female high school students' interest and sense of belonging in STEM courses.

#### Stereotype Threat and Boys' Performance, Motivational Dispositions, and Attitudes

Effects of stereotypes on boys' performance, motivational dispositions, and attitudes toward a domain are less clear, as there are only a few studies on such effects and contradictory findings have been reported. Muzzatti and Agnoli (2007), for example, found no effects of presenting stereotypes on boys' math performance in Grades 3, 5, and 8 as well as their math competence beliefs in Grades 3 and 5 (see also Hermann and Vollmeyer, 2016 for similar results on boys in elementary school). However, among 8th graders, they found higher competence beliefs among boys who were confronted with the stereotype of males' advantage in math compared to the control group (Muzzatti and Agnoli, 2007). Similarly, Master et al. (2015) found no effects of stereotypes on male adolescents' sense of belonging and interest in enrolling in computer courses.

In addition, there is some work on the effects of stereotypes on males using adult samples that also suggest that males are not much affected by stereotypes (Walton and Cohen, 2003; Cheryan et al., 2009; Fogliati and Bussey, 2013; Doyle and Voyer, 2016). Although a meta-analysis by Walton and Cohen (2003) indicated positive effects of traditional gender stereotypes for men's math performance, a more recent meta-analysis by Doyle and Voyer (2016) found no effects. Furthermore, no effects of traditional gender stereotypes have been reported with respect to men's interest and belonging in computer science (Cheryan et al., 2009) or their motivation to improve in math (Fogliati and Bussey, 2013).

In sum, several studies indicate effects of stereotypes on females' performance, motivational dispositions, and attitudes toward math, whereas most studies have reported no effects for males. Nevertheless, the abovementioned studies on stereotype threat effects should be interpreted with caution because the robustness of such effects has recently been called into question due to indications of publication bias in a meta-analysis of this research (Flore and Wicherts, 2015).

### Effects of Stereotypes Presented in the Media

Research on expectancy-value theory has focused primarily on the influence of parents, teachers, or peers on children's endorsement of stereotypes and their expectancy and value beliefs (see Wigfield et al., 2015), but research in the area of media psychology and communication studies has suggested that television programs and movies can contribute to children's gender-role learning in terms of their perceptions of gender-typical occupations (Steinke et al., 2007) or their gender-role values and interpersonal attraction (Aubrey and Harrison, 2004). In addition, research on stereotype threat has indicated a wide range of situations, such as newspaper articles (Cheryan et al., 2013), images in schoolbooks (Good et al., 2010), and photographs (Muzzatti and Agnoli, 2007), in which stereotypes about females' underperformance in math can affect both females and males.

In a recent meta-analysis, Appel and Weber (2017) investigated how stereotypes in mass media (e.g., newspapers, cartoons, advertisements) can affect stereotyped and non-stereotyped groups. In this analysis, negative effects of *d* = −0.38 for members of the stereotyped group and positive effects of *d* = 0.17 for members of the non-targeted group were reported.

Additionally, there are a few studies specifically investigating effects of stereotypes in videos and television advertising (Davies et al., 2002; Murphy et al., 2007; Bond, 2016). Bond (2016) presented short clips of different television shows (about 2 min long) to elementary school girls in a gender stereotype condition, a counter-stereotype condition, and a neutral control condition. No effects of the stereotypes were found on math and science competence beliefs or interest in STEM-related careers. However, girls in the stereotype condition reported more interest in stereotypical careers than those in the other two conditions.

In an adult sample, Murphy et al. (2007) found negative effects of reminding women of their underrepresentation in math-intensive STEM fields via video on their sense of belonging as well as intention to participate in a STEM-related conference. In this study, women in the stereotyped condition watched a video in which the male-female ratio reflected the proportion of women in these fields, whereas women in the control condition watched a video with a gender-balanced proportion.

Davies et al. (2002) showed that women experience stereotype threat when they are reminded of existing stereotypes about women in television advertising. In this study, participants watched commercials in which women were very excited about buying cosmetic products or trying a new baking recipe. After watching these commercials, women performed worse on a math test compared with men who watched the same commercials and compared with women who watched gender-neutral commercials. The results furthermore showed that women preferred verbal tasks and avoided math-related tasks after watching such commercials compared with the control group and men in the experimental group. Women also showed less interest in educational and vocational areas that are typically male-stereotyped but higher interest in typically female-stereotyped domains.

The reported studies indicate that stereotypes in videos can have negative effects on females. However, these findings provide only initial insights into the effects of television. Furthermore, these studies investigated stereotypes that were presented in isolated situations. Thus, they were not able to provide insights into how stereotypes might affect children when experienced in their daily lives in more complex situations, for instance, as one part of a whole television program.

### The Present Study

In the present study, we investigated effects of gender stereotypes in a STEM television program on girls' and boys' stereotype endorsement, their math performance, their motivational dispositions (i.e., expectancy and value beliefs), and their attitudes (i.e., sense of belonging and feeling) toward math. Despite the importance of television programs in children's everyday lives and the relevance of such programs for children's informal science learning, there is a lack of research on how girls' and boys' reception of STEM television programs might be affected in different ways by presentations of traditional gender stereotypes in such programs. Research on expectancy-value theory and stereotype threat has provided initial insights into how stereotypes might affect children. However, research on expectancy-value theory has mainly focused on the role of stereotypes that are conveyed by parents, teachers, or peers (see Wigfield et al., 2015), and research on stereotype threat has traditionally investigated effects of stereotypes presented as isolated stimuli in laboratory settings on adults (see Spencer et al., 2016). Furthermore, there are indications of publication bias in the stereotype threat literature (Flore and Wicherts, 2015). Accordingly, it is unclear whether and how stereotypes embedded in children's daily activities such as in a television program might affect girls and boys.

Therefore, we conducted a randomized study in which fifth-grade students watched a children's television program about math that either contained or did not contain a clip in which traditional gender stereotypes were made salient. We chose this age group because of specific developmental processes in children's expectancy and value beliefs during that age. During their elementary school years, children become increasingly better at understanding, interpreting, and integrating the feedback of others (for a review, see Wigfield et al., 2015). Therefore, they become more realistic in evaluating their own strengths and weaknesses during that period and link their expectancy and value beliefs more closely to environmental experiences than younger elementary school children (for a review, see Wigfield et al., 2015). Additionally, children become increasingly aware of social gender roles and how behavior might reflect such roles (for a review, see Leaper, 2015). In order to link the study as closely as possible to what children are likely to watch in their everyday lives, we used a television program that was broadcast on a national TV channel in Germany as the experimental material. The chosen program was designed to show children that math could be interesting and fun and included a section with stereotypes in which two girls were frustrated that they had to do math and then decided to copy their homework from a male classmate.

According to expectancy-value theory, experiencing gender stereotypes leads girls to disidentify with math and devalue the subject, whereas boys may particularly identify with and value math. As a result of such processes, boys develop higher competence beliefs and values in male-typed domains such as math and math-intensive STEM domains than girls (e.g., Wigfield et al., 2015). In order to obtain a comprehensive picture of how stereotypes can affect such socialization processes, we examined effects of the experimental manipulation on different outcomes. First, we explored how the stereotypes affect children's stereotype endorsement. Second, we examined effects on sense of belonging in math and feeling toward the domain as indicators of children's identification with the subject. Third, we investigated effects on self-concept (as an indicator of expectancy beliefs), the four task values as well as performance in math. We pre-registered our predictions on the effects for these outcomes before conducting the experiment in order to increase research transparency (https://osf.io/8f7y6/?view_only=d85b73e70f5040b5a54fcf03091811f1). As such, we followed the recommendations of Wagenmakers et al. (2012) and van't Veer and Giner-Sorolla (2016) by pre-registering hypotheses and exploratory research questions as well as information on the experimental design, the sample, the variables, and the analysis strategy.

On the basis of existing literature on effects of stereotypes on math performance (Flore and Wicherts, 2015), self-concept (Cadinu et al., 2003; Muzzatti and Agnoli, 2007), and sense of belonging (Master et al., 2015), we expected that girls who watched the gender-stereotyped television program would show lower math performance, lower math self-concept, and a lower sense of belonging in math compared with girls in the control condition.

We explored effects on girls' task values in math and their feelings about math as open-ended research questions. There is only sparse evidence on how task values might be influenced by gender stereotypes (Plante et al., 2013; Smith et al., 2015), and previous work has not differentiated between the four components (intrinsic value, attainment value, utility value, and cost). Furthermore, to the best of our knowledge, there is no work that has investigated effects of stereotypes on children's feelings about a domain. We therefore did not hypothesize specific effects on task values and feelings about math.

In order to gain insights into possible differential effects of such stereotypes on girls and boys, we explored effects on boys' performance, expectancy and value beliefs, sense of belonging and feeling toward the domain in math-related constructs as well, using the same outcomes measures. Due to the mixed findings from previous research on the effects of stereotypes on such constructs for males, we did not hypothesize specific effects for boys but rather investigated possible effects on these outcomes for boys as exploratory research questions.

We did not formulate any specific hypotheses with respect to the endorsement of gender stereotypes among both girls and boys, because previous research has provided mixed results on the effects of gender stereotypes on children's endorsement of gender stereotypes (Ambady et al., 2001; Schmader et al., 2004; Steffens et al., 2010).

## Methods

### Participants

Participants were 335 fifth-grade students. Children were recruited from 18 classes of four academic track schools (Gymnasium) in Baden-Württemberg, Germany. The sample size was based on a power analysis for a randomized block trial with the treatment implemented at the student level using Optimal Design (Raudenbush et al., 2011). We calculated the required number of classrooms by aiming to achieve an acceptable level of power (β = 0.80) to detect medium-sized intervention effects (δ = 0.40) when comparing the experimental with the control condition. We assumed that 10 girls and 10 boys would participate in each class, and they would be randomly assigned to the control and experimental conditions. We furthermore assumed an effect size variability of 0.10 (for more details, see the preregistration protocol).

Children participated in the study on a voluntary basis, and for every participant, we obtained written consent from a parent. The mean age of the sample was 10.08 years (*SD* = 0.38), and the number of girls and boys who participated in the study was almost equal (48.7% girls).

### Design and Procedure

As preregistered, we collected the data using a pretest–posttest design, and we applied a randomized block design to examine effects of gender stereotypes in a television program. Girls and boys were randomly assigned to the experimental and control conditions within each class (experimental condition: *N* = 87 girls and *N* = 85 boys; control condition: *N* = 76 girls and *N* = 87 boys). Participants were tested in one classroom simultaneously, but every student watched the video separately on an iPad with headphones. We collected the pretest data 1 week before the experimental manipulation and the posttest data directly after the experimental manipulation. The presentation order of the achievement test and the questionnaire was balanced on the class level in both phases of data collection because research on stereotype threat has shown that even small and short manipulations can influence students' performance, motivational dispositions, and attitudes (e.g., Master et al., 2015; i.e., the achievement test might affect students' motivational dispositions and attitudes if assessed first, or the questionnaire might wash out any effects on performance). We randomly assigned the classes to these two conditions (*N* = 9 classes in each condition). Data were collected in June and July 2016 by trained research assistants during school hours (a maximum of one lesson for the pretest, a maximum of two lessons for the experiment and the posttest).

### Experimental Manipulation

As experimental material, we used one episode from a German children's television program, which was broadcast on a German national television channel in June 2015. The episode focused on math and was designed to show children that math could be interesting and fun even though it might be experienced as boring in school (KiKa.de, 2015). The episode had a total duration of 23 min. As preregistered, only 15 min of the episode were used in the present study due to time constraints. This included an introduction by a male television presenter (about 1 min) and two different math tasks solved by fifth-grade children (about 13 min). In addition, the video included a clip that implied traditional gender stereotypes in math (about 1 min). This part showed two girls who were very frustrated that they had to do math homework. Instead of doing their homework, one girl copied it from a male classmate, and in exchange, she promised him that her friend would accompany him to the movies. Her friend was horrified about going out with this boy because he seemed rather geeky. He was wearing very large glasses, a shirt that was completely buttoned up, suit trousers, and suspenders. Such stereotypes of the geeky math boy are often presented in movies or television programs (see e.g., Heyman, 2008; Collins, 2011).

The introduction and the math tasks solved by the children were used in both conditions. The experimental manipulation depended on only the last minute of the video. In the experimental condition, participants watched the gender-stereotyped clip. In the control condition, participants watched a neutral summary of the first 14 min of the video. The summary was comparable in length so that the total length of the video would be held constant between the conditions. Consequently, participants experienced the stereotype as a short section within the whole television program so that the ecological validity of the experiment would be high.

Because the television program was broadcast on a national TV channel in Germany, we assessed whether participants had already seen the video beforehand, which was the case for 41 students. As a robustness check, we computed all analyses without these students, but the results did not differ meaningfully (see the Supplemental Material).

### Instruments

We used an achievement test and a questionnaire to assess effects of the experimental manipulation. The instruments were identical at pre- and posttest, with the exception of questions about the video, which were only assessed at posttest.

#### Math Performance

We assessed students' math performance with a speed test that consisted of three sections containing basic tasks involving addition, subtraction, and multiplication (basic competence test; Lambert et al., in preparation). Each part consisted of 36 tasks, and for each individual part, we asked the students to solve as many tasks as possible within 2 min. The sum score of all three parts, generated by computing the sum of correctly solved items, was used in the analyses. The test showed high internal consistency (Kuder-Richardson 20 = 0.93/0.94 for the pretest/posttest).

#### Questionnaire

We assessed children's stereotype endorsement, their motivational dispositions (i.e., self-concept and value beliefs) as well as their attitudes toward math (i.e., sense of belonging and feelings) with a questionnaire to capture whether children (dis)identify with and (de)value this domain after watching the video including the stereotypes. Unless otherwise noted, all items on the questionnaire were measured with a 4-point Likert scale ranging from 1 (*completely disagree*) to 4 (*completely agree*). The 4-point Likert scale was used to avoid confounding response factors in scales containing a middle category (Kaplan, 1972; Dubois and Burns, 1975). Additionally, four response options seems to be optimal for children, as they are not able to differentiate between more categories (Borgers et al., 2004). Due to the small number of response options, we carefully checked the degree of non-normality in our data. Although there was some variation across scales, the skewness and kurtosis values all fell within an acceptable range (average skewness was −0.36, with no scale having a skewness >1.4, and the average kurtosis was 0.59, with only 2 scales having a kurtosis >1). The questionnaire is available at https://osf.io/8f7y6/?view_only=d85b73e70f5040b5a54fcf03091811f1.

##### Stereotype endorsement

We assessed stereotype endorsement with three items based on items from Schmader et al. (2004). We adapted the items for children by using “boys” and “girls” in the wording instead of “men” and “women” (e.g., “Boys have higher math abilities than girls”; α = 0.76/0.76 for the pretest/posttest).

We extended the scale by including two items in which the words “boys” and “girls” were interchanged (e.g., “Girls have better math abilities than boys”) and preregistered this extension. We recoded these items before computing the scale score. Because the reliability of the extended scale was rather low (α = 0.52/0.55 for the pretest/posttest), we used only the original scale in our analyses.

##### Task values

We assessed students' value beliefs in math with scales from Gaspard et al. (2015). The items covered all four conceptual dimensions of task values as specified in the expectancy-value model (Wigfield and Eccles, 2000). Intrinsic value (e.g., “I like doing math”; α = 0.92/0.94 for the pretest/posttest), attainment value (e.g., “It is important to me to be good at math”; four items; α = 0.87/0.93 for the pretest/posttest), and cost (emotional costs, e.g., “Studying math makes me quite nervous”; α = 0.78/.86 for the pretest/posttest) were assessed with four items each. For utility value, we differentiated between two facets: utility for daily life (e.g., “Knowing about the subject of math brings me many advantages in my daily life”; α = 0.82/0.84 for the pretest/posttest) and social utility (e.g., “Sound knowledge in math counts for something with my classmates”; α = .68/.80 for the pretest/posttest), which were both assessed with three items.

##### Self-concept

We assessed self-concept with a math self-concept scale comprised of four items (e.g., “I am good at math”; α = .86/.86 for the pretest/posttest), which has been well-validated in previous studies (see Gaspard et al., 2016).

##### Sense of belonging

We assessed students' sense of belonging in math with 10 items (e.g., “I feel like a real part of my class in math”), based on the Psychological Sense of School Membership (PSSM; Goodenow, 1993). The items were translated into German and adapted to math class instead of school membership. Due to low item-scale correlations (*r*_*it*_ = 0.03/0.16 for the pretest/posttest), we excluded 1 item when we computed the scale. The final scale therefore consisted of 9 items and showed an acceptable internal consistency (α = 0.76/0.84 for the pretest/posttest). Because we did not preregister the exclusion of the item, we conducted the analysis for this outcome also using the original scale, which included all 10 items. The internal consistency for this scale was acceptable (α = 0.73/0.83 for the pretest/posttest), and the results did not differ meaningfully from those computed with the reduced scale (see the Supplemental Material for this as well as for model fit indices from confirmatory factor analyses of the scales).

##### Explicit attitudes toward math

We assessed explicit attitudes toward math with a feeling thermometer as used by Kessels et al. (2006). Students were asked to rate their preferences using scales ranging from 0 (*cold/unfavorable*) to 100 (*warm/favorable*) for math and German. As done by Kessels et al. (2006), we calculated the difference between the two scores as an indicator of students' attitudes toward the domains. Therefore, the final score consisted of possible values ranging from −100 to +100, whereby positive values indicated positive attitudes toward math relative to German, and negative values indicated negative attitudes toward math relative to German.

##### Additional scales

As preregistered, we additionally assessed stereotype endorsement with measures based on studies by Ambady et al. (2001) and Steffens et al. (2010) in which the participants were asked how much they would like to engage in activities related to math and German. Due to high rates of missing data and the low reliability of these scales, we refrained from conducting additional analyses on these instruments.

We furthermore preregistered analyses with respect to the same set of constructs (i.e., task values, self-concept, sense of belonging) in the domain of German. Dimensional comparisons of complementary domains are important in the development of students' motivational dispositions (Möller and Marsh, 2013), and there are initial findings on how motivational dispositions in a verbal domain might be affected by traditional gender stereotypes in commercials (Davies et al., 2002). Due to space limitations, the results on girls' and boys' motivational dispositions and attitudes in German are reported in the Supplemental Material. In summary, we found no effects of the experimental condition on girls' and boys' motivational dispositions and attitudes in German except that girls in the experimental condition reported lower cost in German than those in the control condition.

### Statistical Analyses

In order to estimate effects of the gender stereotypes in the television program, we computed multiple regression analyses for the different outcomes in Mplus 7.31 (Muthén and Muthén, 2012) as preregistered. All models included student gender (pacifier coded, boy = 1), the experimental condition (a pacifier-coded variable based on students' original assignment, experimental condition = 1), and the Gender × Condition interaction as predictor variables. In addition, we included the respective pretest measures as covariates to estimate the effect of the experimental manipulation more precisely (Raudenbush, 1997). In order to make it easier to interpret the results, we standardized all continuous predictors (i.e., the pretest scores) and the respective dependent variable.

In our analyses, we conducted an intention-to-treat analysis by taking only the original assignment into account in order to keep the randomization to the experimental and control conditions intact (Shadish et al., 2002). As a robustness check, we ran all analyses without the students who did not correctly answer a question about what they had seen in the last minute of the video, that is, two girls who copied the homework of a classmate in the experimental condition or a summary of the video in the control condition (*n* = 13). This question was assessed at the end of the posttest questionnaire. The results did not differ meaningfully and are presented in the Supplemental Material.

To test whether there were any order effects of the instruments, we computed multiple-group regression analyses with the order of the instruments as the grouping variable. We tested the difference between the models for each group with Wald χ^2^ tests. If there were no significant differences between the coefficients in the models, we calculated multiple regressions for the whole sample.

Missing data ranged from 2.1% to 9.9% for the different scales because some students were absent when the pre- or post-test was given, and some students did not respond to individual scales. To deal with missing data, we used the full information maximum likelihood approach as implemented in Mplus 7.31 (Muthén and Muthén, 2012).

We considered the clustered structure of the data (students nested in classes) by using the design-based correction of standard errors implemented in Mplus 7.31 (Muthén and Muthén, 2012).

## Results

### Descriptive Statistics and Randomization Check

The means and standard deviations for all scales are shown by gender and condition in Tables 1–3. Compared with boys, girls showed significantly lower math performance and reported lower levels of the feeling thermometer, self-concept, intrinsic value, and social utility value on the pretest. The correlations for the outcome variables indicate that the mean levels were relatively stable across the two measurement points for all outcomes (0.60 < *r* < 0.87; see Table 4).

**Table 1:** Descriptive statistics for all study variables on the pretest separated by gender.

**Variable**	**Girls**		**Boys**		***d*^a^**	***d*** **95% CI**	
	***M***	***SD***	***M***	***SD***		
Stereotype endorsement T1	2.55	0.52	2.73	0.45	0.35	0.20	0.50
Performance T1	51.88	8.14	56.09	8.99	0.48	0.33	0.62
Self-concept T1	3.15	0.73	3.40	0.61	0.37	0.20	0.55
Sense of belonging T1	3.16	0.48	3.19	0.45	0.05	−0.16	0.26
Feeling thermometer T1	1.62	33.48	15.80	32.07	0.42	0.25	0.60
Intrinsic value T1	3.12	0.76	3.27	0.74	0.20	0.03	0.37
Attainment value T1	3.50	0.57	3.46	0.61	−0.06	−0.31	0.20
Utility value—daily life T1	3.24	0.66	3.26	0.68	0.02	−0.15	0.20
Utility value—social T1	2.22	0.68	2.41	0.64	0.29	0.11	0.47
Cost T1	1.60	0.60	1.53	0.52	−0.13	−0.33	0.08

*C = confidence intervall*.

a *The dependent variable is standardized*.

**Table 2:** Descriptive statistics for all outcome variables at T1 separated by gender and condition.

	**Girls**								**Boys**							
	**Experimental condition**				**Control condition**				**Experimental condition**				**Control condition**			
**Variable**	***M***	***SD***	**Min**	**Max**	***M***	***SD***	**Min**	**Max**	***M***	***SD***	**Min**	**Max**	***M***	***SD***	**Min**	**Max**
Stereotype endorsement T1	2.52	0.47	1.00	3.33	2.59	0.58	1.00	4.00	2.70	0.44	1.67	3.67	2.76	0.47	2.00	4.00
Performance T1	51.33	8.28	33.00	71.00	52.51	7.98	31.00	73.00	55.79	8.65	34.00	74.00	56.40	9.38	37.00	86.00
Self-concept T1	3.20	0.64	1.25	4.00	3.09	0.75	1.50	4.00	3.39	0.61	1.50	4.00	3.41	0.61	1.75	4.00
Sense of belonging T1	3.16	0.51	1.63	4.00	3.16	0.46	1.57	4.00	3.10	0.47	1.56	3.89	3.27	0.43	2.11	4.00
Feeling thermometer T1	3.86	34.10	−80.00	100.00	−0.88	32.83	−100.00	90.00	16.40	35.84	−90.00	100.00	15.19	27.89	−70.00	70.00
Intrinsic value T1	3.16	0.74	1.00	4.00	3.07	0.77	1.00	4.00	3.28	0.74	1.00	4.00	3.26	0.75	1.00	4.00
Attainment value T1	3.54	0.49	2.00	4.00	3.45	0.65	1.00	4.00	3.46	0.62	1.50	4.00	3.46	0.60	1.50	4.00
Utility value—daily life T1	3.20	0.64	1.33	4.00	3.29	0.67	1.33	4.00	3.20	0.67	1.00	4.00	3.32	0.68	1.00	4.00
Utility value—social T1	2.22	0.65	1.00	4.00	2.22	0.71	1.00	4.00	2.41	0.61	1.00	3.67	2.42	0.67	1.00	4.00
Cost T1	1.58	0.55	1.00	3.50	1.63	0.66	1.00	4.00	1.53	0.51	1.00	3.00	1.53	0.53	1.00	3.50

**Table 3:** Descriptive statistics for all outcome variables at T2 separated by gender and condition.

	**Girls**								**Boys**							
	**Experimental condition**				**Control condition**				**Experimental condition**				**Control condition**			
**Variable**	***M***	***SD***	**Min**	**Max**	***M***	***SD***	**Min**	**Max**	***M***	***SD***	**Min**	**Max**	***M***	***SD***	**Min**	**Max**
Stereotype endorsement T2	2.68	0.51	1.00	4.00	2.45	0.56	1.00	3.67	2.75	0.50	1.00	4.00	2.67	0.48	1.67	4.00
Performance T2	54.52	8.42	31.00	73.00	55.28	8.40	36.00	73.00	58.04	8.04	39.00	73.00	58.74	8.19	37.00	73.00
Self-concept T2	3.21	0.66	1.00	4.00	3.08	0.76	1.00	4.00	3.43	0.62	1.75	4.00	3.33	0.61	1.75	4.00
Sense of belonging T2	3.12	0.54	1.56	4.00	3.18	0.53	1.78	4.00	3.12	0.50	1.67	4.00	3.15	0.55	1.67	4.00
Feeling thermometer T2	1.46	35.04	−100.00	100.00	1.00	36.00	−100.00	100.00	18.00	33.22	−80.00	90.00	17.60	32.50	−70.00	100.00
Intrinsic value T2	3.16	0.79	1.00	4.00	3.05	0.86	1.00	4.00	3.26	0.73	1.00	4.00	3.17	0.75	1.50	4.00
Attainment value T2	3.57	0.56	2.00	4.00	3.53	0.62	1.25	4.00	3.49	0.60	1.25	4.00	3.52	0.62	2.00	4.00
Utility value—daily life T2	3.36	0.59	1.33	4.00	3.40	0.63	1.00	4.00	3.29	0.68	1.00	4.00	3.37	0.68	1.33	4.00
Utility value—social T2	2.22	0.71	1.00	4.00	2.06	0.70	1.00	4.00	2.20	0.72	1.00	3.67	2.32	0.78	1.00	4.00
Cost T2	1.54	0.65	1.00	4.00	1.63	0.70	1.00	4.00	1.47	0.53	1.00	3.00	1.49	0.57	1.00	3.50

**Table 4:** Correlations between all Study Variables.

	**Variable**	**1**	**2**	**3**	**4**	**5**	**6**	**7**	**8**	**9**	**10**	**11**	**12**	**13**	**14**	**15**	**16**	**17**	**18**	**19**	**20**
1	Stereotype endors. T1	—																			
2	Stereotype endors. T2	0.38	—																		
3	Performance T1	(0.11)	(0.09)	—																	
4	Performance T2	0.13	0.15	0.85	—																
5	Self-concept T1	(0.09)	(0.08)	0.36	0.35	—															
6	Self-concept T2	0.12	0.11	0.30	0.33	0.80	—														
7	Sense of belonging T1	(0.10)	(0.10)	0.14	0.19	0.49	0.49	—													
8	Sense of belonging T2	(0.05)	(0.09)	0.12	0.17	0.39	0.50	0.81	—												
9	Feeling thermo. T1	(0.07)	(0.01)	0.19	0.22	0.48	0.44	0.33	0.29	—											
10	Feeling thermo. T2	0.11	(0.04)	0.20	0.26	0.42	0.45	0.32	0.28	0.87	—										
11	Intrinsic value T1	0.11	(0.09)	0.15	0.18	0.75	0.72	0.53	0.46	0.51	0.51	—									
12	Intrinsic value T2	(0.05)	(0.07)	0.18	0.22	0.68	0.76	0.46	0.47	0.39	0.41	0.87	—								
13	Attainment value T1	(0.10)	(0.07)	(−0.05)	(−0.00)	0.29	0.22	0.30	0.25	0.22	0.17	0.33	0.25	—							
14	Attainment value T2	(0.06)	(0.05)	(−0.01)	(0.03)	0.24	0.27	0.28	0.32	0.18	0.19	0.29	0.32	0.70	—						
15	Utility v: daily life T1	0.15	(0.08)	(−0.06)	(−0.03)	0.25	0.22	0.32	0.26	0.23	0.21	0.38	0.30	0.42	0.33	—					
16	Utility v: daily life T2	(0.06)	(−0.01)	(−0.10)	(−0.07)	0.16	0.22	0.30	0.36	(0.11)	0.15	0.30	0.33	0.30	0.45	0.60	—				
17	Utility v: social T1	0.16	(0.07)	(0.10)	0.16	0.34	0.28	0.36	0.31	0.26	0.26	0.39	0.34	0.43	0.29	0.31	0.23	—			
18	Utility v: social T2	0.13	(0.03)	(0.09)	(0.07)	0.24	0.27	0.25	0.28	0.21	0.24	0.35	0.33	0.34	0.28	0.23	0.24	0.70	—		
19	Cost T1	(−0.01)	(−0.06)	−0.16	−0.19	−0.68	−0.68	−0.53	−0.47	−0.42	−0.36	−0.71	−0.68	−0.16	−0.18	−0.23	−0.21	−0.17	(−0.10)	—	
20	Cost T2	(−0.01)	(−0.05)	−0.11	−0.19	−0.59	−0.73	−0.45	−0.51	−0.31	−0.31	−0.56	−0.68	(−0.09)	−0.23	−0.18	−0.25	−0.15	(−0.10)	0.72	—

*Nonsignificant correlations are displayed in parentheses; for all other correlations, p < 0.05*.

*Stereotype endors. = Stereotype endorsement; Feeling thermo. = Feeling thermometer; Utility v = Utility value*.

To test whether the randomization in the two conditions had been successful in the baseline measures, we computed multiple regression models as preregistered (pretest values regressed on the experimental condition, gender, and the Gender × Condition interaction). There were no significant differences between the conditions for girls and boys on the pretest values for all variables (all *p*s > 0.137) except for the boys with respect to sense of belonging. Here, boys in the experimental condition showed lower baseline scores than those in the control condition [*d* = 0.36, 95% CI [0.07, 0.65]]. As preregistered, we controlled for the pretest scores in all analyses to estimate the effect of the experimental manipulation more precisely because of the explanatory power of this covariate.

### Effects of the Experimental Manipulation

First, we tested if there were any order effects of the instruments by computing multiple-group regression analyses using the order of the instruments as the grouping variable. Wald χ^2^ tests indicated no differences in these models with respect to any of the studied outcomes (all *p*s > 0.154) except for social utility value, where the coefficients for the Gender × Condition interaction differed significantly, $\chi_{\left(1\right)}^{2}$ = 11.76, *p* = 0.001. Consequently, we computed multiple regression analyses using the total sample for all outcomes (i.e., averaged across instrument order) except for social utility value (see Tables 5–7).

**Table 5:** Multiple regression models 1: effects on stereotype endorsement, performance, self-concept, sense of belonging, and feeling thermometer.

**Predictor**	**Stereotype endorsement**		**Performance**		**Self-concept**		**Sense of belonging**		**Feeling thermometer**	
	**β**	**β 95% CI**	**β**	**β 95% CI**	**β**	**β 95% CI**	**β**	**β 95% CI**	**β**	**β 95% CI**
Pretest	0.39^***^	[0.26, 0.52]	0.86^***^	[0.81, 0.91]	0.81^***^	[0.73, 0.89]	0.81^***^	[0.75, 0.87]	0.86^***^	[0.80, 0.92]
Gender (boys = 1)	0.29^†^	[0.03, 0.55]	0.10	[−0.06, 0.26]	−0.01	[−0.14, 0.11]	−0.20^*^	[−0.36,−0.04]	0.04	[−0.10, 0.18]
Condition (exp. = 1)	0.50^***^	[0.03, 0.74]	0.04^a^	[−0.10, 0.18]	0.03^a^	[−0.09, 0.15]	−0.10^a^	[−0.23, 0.02]	−0.12^†^	[−0.23, −0.01]
Gender × Condition	−0.28	[−0.58, 0.02]	−0.07	[−0.24, 0.09]	0.12	[−0.03, 0.26]	0.30^**^	[0.12, 0.49]	0.10	[−0.07, 0.27]
Effect of condition for boys	0.22^*^	[0.04, 0.40]	−0.03	[−0.15, 0.09]	0.14^†^	[0.00, 0.29]	0.20^*^	[0.04, 0.36]	−0.02	[−0.13, 0.10]

*All continuous variables are standardized. CI = confidence interval; exp. = experimental condition*.

a *We formulated a hypothesis for this effect prior to the analysis*.

† *p < 0.10*.

* *p < 0.05*.

** *p < 0.01*.

*** *p < 0.001*.

**Table 6:** Multiple regression models 2: effects on intrinsic value, attainment value, utility value for daily life, and cost.

**Predictor**	**Intrinsic value**		**Attainment value**		**Utility value: daily life**		**Cost**	
	**β**	**β 95% CI**	**β**	**β 95% CI**	**β**	**β 95% CI**	**β**	**β 95% CI**
Pretest	0.87^***^	[0.80, 0.93]	0.71^***^	[0.62, 0.79]	0.62^***^	[0.52, 0.71]	0.71^***^	[0.62, 0.80]
Gender (boys = 1)	−0.03	[−0.17, 0.11]	−0.05	[−0.19, 0.09]	−0.09	[−0.32, 0.15]	−0.11	[−0.35, 0.12]
Condition (exp. = 1)	0.05	[−0.10, 0.19]	−0.02	[−0.14, 0.10]	0.03	[−0.17, 0.23]	−0.05	[−0.21, 0.11]
Gender × Condition	0.00	[−0.18, 0.18]	0.00	[−0.21, 0.21]	−0.03	[−0.31, 0.26]	0.05	[−0.19, 0.30]
Effect of condition for boys	0.05	[−0.09, 0.18]	−0.02	[−0.18, 0.15]	0.00	[−0.19, 0.19]	0.00	[−0.23, 0.23]

*All continuous variables are standardized. CI = confidence interval; exp. = experimental*.

*** *p < 0.001*.

**Table 7:** Multiple-group multiple regression model: effects on social utility value.

**Predictor**	**Utility value—social**			
	**Questionnaire first**		**Achievement test first**	
	**β**	**β 95% CI**	**β**	**β 95% CI**
Pretest	0.65^***^	[0.60, 0.71]	0.76^***^	[0.70, 0.82]
Gender (boys = 1)	0.30^**^	[0.14, 0.47]	0.10	[−0.13, 0.34]
Condition (exp. = 1)	0.24^†^	[0.00, 0.48]	0.21^†^	[0.03, 0.40]
Gender × Condition	−0.88^***^	[−1.12, −0.64]	−0.08	[−0.38, 0.22]
Effect of condition for boys	−0.64^**^	[−0.98, −0.30]	0.14	[−0.05, 0.32]

*All continuous variables are standardized. CI = confidence interval; exp. = experimental*.

† *p < 0.10*.

** *p < 0.01*.

*** *p < 0.001*.

We specified multiple regressions to test effects of the experimental manipulation (see Tables 5, 6). As girls were coded 0, the main effect of the experimental condition was equal to the simple slope for girls, whereas the Gender × Condition interaction term indicated whether the effects differed between boys and girls. Because we were more interested in investigating effects of the experimental manipulation on girls' and boys' performance, motivational dispositions, and attitudes rather than on gender differences in these outcomes, we additionally estimated the simple slopes for boys for all outcomes using the model constraint in Mplus.

With respect to stereotype endorsement, we did not hypothesize specific effects due to mixed previous results for effects of stereotype threat on this outcome. The results revealed a significant positive effect of the experimental condition for girls. The same result held for boys because the Gender × Condition interaction was not statistically significant (see Table 5).

**Figure 1:**
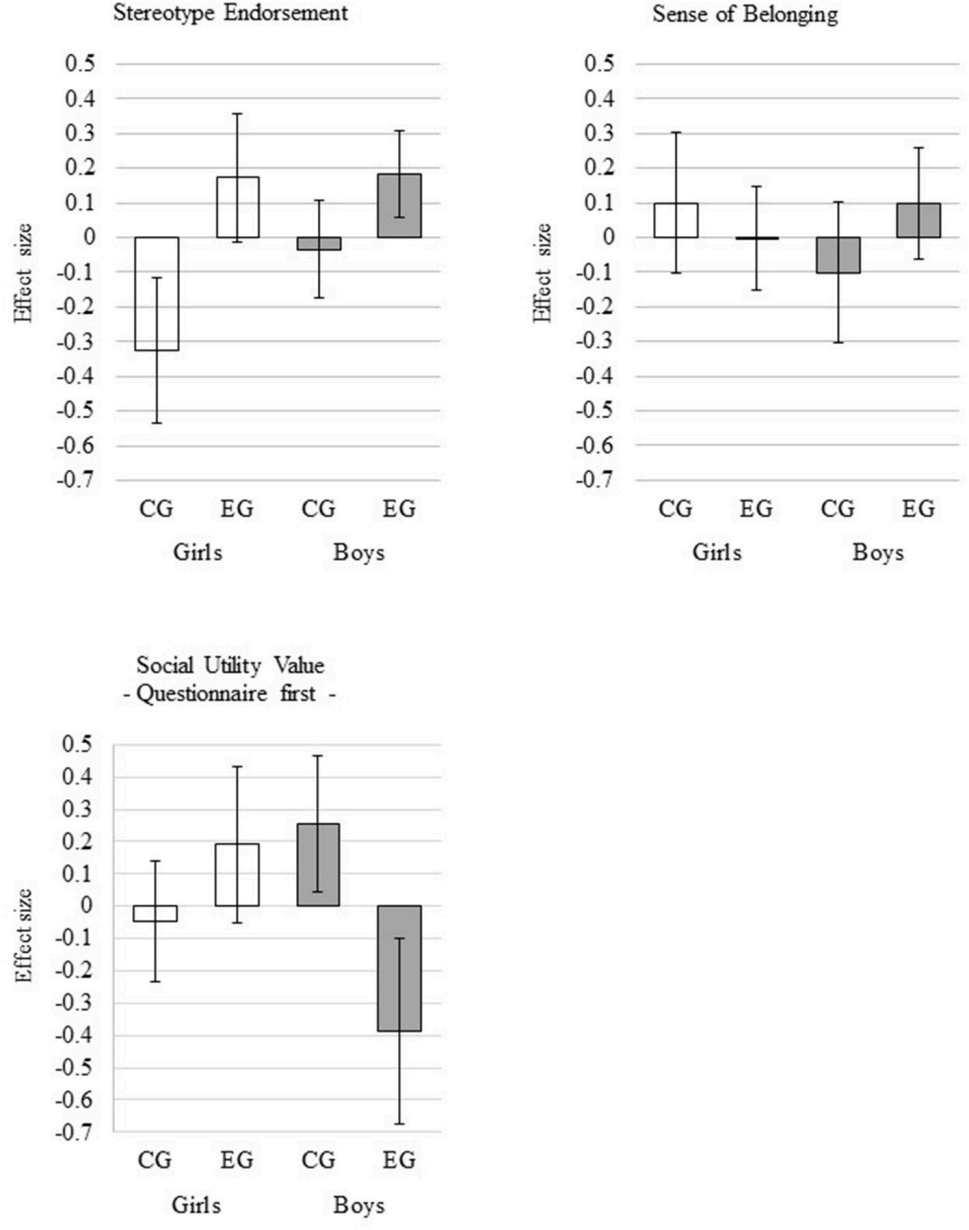
Effects of the experimental manipulation. Error bars represent 95% confidence intervals. CG = control group; EG = experimental group.

Regarding math performance, math self-concept, and sense of belonging, we hypothesized that girls in the experimental condition would score lower on these outcomes than girls in the control condition. For boys, we did not hypothesize specific effects. For these outcomes, the results revealed no significant effect of the experimental condition for girls. For math performance and math self-concept, there were also no effects of the condition for boys. With respect to sense of belonging, the Gender × Condition interaction was statistically significant, and there was a positive effect of the condition for boys, indicating that in contrast to girls, boys in the experimental condition showed higher values of sense of belonging than boys in the control condition (see Table 5 and Figure 1).

Regarding task values and attitudes toward math assessed with the feeling thermometer, we did not hypothesize specific effects of the experimental condition for girls and boys. With respect to the feeling thermometer, intrinsic value, attainment value, utility value for daily life, and cost, we found no significant effects of the experimental condition for either girls or boys (see Table 6).

For social utility, we computed multiple-group regression analyses using the order of the instruments as a grouping variable because a Wald χ^2^ test indicated effects of the order of the instruments in the assessment as described above. Because we were interested in the effects of the experimental manipulation on social utility assessed with the questionnaire, the results for the students who were given the questionnaire first in the assessment were of major interest. For the students who were given the questionnaire first, there was no significant effect of the condition for girls, but the Gender × Condition interaction was statistically significant, indicating that boys in the experimental condition reported a significantly lower social utility score than those in the control condition (see Table 7 and Figure 1). For the students who were given the achievement test first, there was no significant effect of the condition for girls or for boys (see Table 7).

## Discussion

In this experimental study, we examined how stereotypes embedded in a children's television program about math influence girls' and boys' stereotype endorsement, math performance, motivational dispositions and attitudes in math. We used a randomized study with a pretest–posttest design and a relatively large sample size, which enabled us to detect medium-sized effects. The material we chose was a television program that had been broadcast on a German national television channel, thus contributing to the high validity of the study. Television programs play a central role in children's everyday lives and are an important part of their informal science learning, but such programs can provide specific gender stereotypes about math (National Reserach Council., 2009; Collins, 2011; Rideout, 2015). Previous research has indicated that the stereotypes children encounter in their environment can impact young girls' and boys' math performance, motivational dispositions, and attitudes. Yet, such research has primarily been conducted in laboratory settings where stereotypes have been presented as isolated stimuli, rather than integrated into other information as would be the case in children's daily lives, for instance, in television programs.

Overall, our results did not indicate that children's performance, motivational dispositions, and attitudes were strongly affected by the stereotypes presented in one part of a television program. However, girls and boys in the experimental condition reported a higher endorsement of stereotypes compared with the respective control condition. Furthermore, boys showed a higher sense of belonging but lower social utility after watching the video that included the stereotypes compared with boys in the control condition. We did not find any effects on either the other motivational dispositions, attitudes or math performance for boys. We also did not find any effects on math performance, motivational dispositions, and attitudes for girls.

### Discussion of the Findings

First of all, the small number of significant effects found in this study support previous research indicating that the short-term effects of stereotypes on performance, motivational dispositions, and attitudes are not as robust as sometimes claimed. For example, Stoet and Geary (2012) reviewed replication attempts of the stereotype threat effect on women's math performance that was found in Spencer et al. (1999) original study. According to this review, only 30% of replication studies confirmed the original finding. In addition, Flore and Wicherts (2015) found indications of publication bias in their meta-analysis on stereotype threat effects in children. In accordance with these findings, the non-significant effects found in our study indicate that stereotype threat effects might occur only in specific situations or might apply only to some children. Here, the negative effect on boys' social utility might add to this discussion because this effect was found only for students who were given the questionnaire first (in the assessment in which we also assessed social utility). We did not find any effects of condition among boys who were given the questionnaire after the achievement test. Therefore, the stereotypes might have affected boys' social utility in the short term, but were washed out after they completed the achievement test, indicating that even if stereotype threat effects occur, they might be very limited in duration and sensitive to other influences.

Nevertheless, specific characteristics of the present study could have also contributed to the small number of effects found. For example, the duration and frequency of the stereotypes presented in the video provide one possible explanation for the fact that we found hardly any effects on girls' and boys' performance, motivational dispositions, and attitudes even though we found an effect on their stereotype endorsement. According to expectancy-value theory, it is through repeated experience that effects begin to accumulate and can lead to the internalization of gender-role stereotypes and to gender differences in expectancy and value beliefs in math in the end (Wigfield and Eccles, 2000; Eccles, 2009). In our study, we used a television program that was broadcast on national television to ensure that the experimental material was strongly linked to children's daily life experiences. However, the stereotyped clip in this television program had a duration of only about 1 min, and the children in the experimental condition saw this clip only once. Thus, the duration and frequency of stereotype presentation might need to be increased in future studies to substantially affect girls' and boys' motivational dispositions.

Furthermore, when interpreting the results of the present study for girls and for boys, the specific age group of the participants should be taken into consideration. We investigated how stereotypes in a television program affect 5th graders because important processes in the development of children's expectancy and value beliefs and understanding of gender role behavior take place during that age period. Around the age of 10 years old, children become increasingly aware of how gender-stereotypical behavior might reflect social gender roles (for a review, see Leaper, 2015). In addition, children increasingly understand, interpret and integrate others' feedback and become more realistic in evaluating their strengths and weaknesses during their elementary school years (Wigfield et al., 2015). Such processes are believed to influence the development of children's expectancy and value beliefs (Wigfield et al., 2015).

We investigated how stereotypes experienced in the environment might influence students' motivational dispositions among 5th graders because children at that age should be right at the beginning of these developmental processes. In addition, previous research has indicated that even elementary school children can be affected by gender stereotypes—at least with respect to math performance (Flore and Wicherts, 2015). However, the participants' young age could be a reason why we found (almost) no effects on students' expectancy and value beliefs. One reason for this assumption is provided by findings from the stereotype threat literature that have indicated that group and domain identification moderate effects of stereotype threat (e.g, Schmader, 2002; Lewis and Sekaquaptewa, 2016). Given that children increasingly identify with specific school subjects in elementary and middle school but do not differentiate much between the subjects at younger ages (see Wigfield et al., 2015), the participants in our study might have been too young and might not have sufficiently identified with the domain of math.

In addition, the stereotypes that were displayed in the video may provide an explanation for the fact that we did not find any effects on girls' motivational dispositions, attitudes, and performance in math and only a few effects on boys' motivational dispositions and attitudes. With respect to the girls in the video, it was not clear whether the girls in the video thought doing their math homework was boring or whether they were not able to solve the problems; thus, the video might have targeted the low motivation of these girls and not their low performance or talent in math, which has typically been the focus of studies that have investigated the effects of stereotype threat (see e.g., Nguyen and Ryan, 2008).

A video that more directly targets girls' lower performance or talent compared with boys might thus evoke stronger effects on girls' motivational dispositions and attitudes. Such a video might also evoke more positive effects on boys' motivational dispositions and attitudes, effects that would go against previous research that has indicated the experience of *stereotype lift* for male students in situations in which female students' disadvantage in math was made salient. Stereotype lift describes the effect of a boost for the non-targeted group in settings in which stereotypes are activated (e.g., for men after negative stereotypes of women's math performance have been presented; e.g., Walton and Cohen, 2003; Johnson et al., 2012). The positive effect on boys' sense of belonging could be an indication of effects of stereotype lift on this outcome due to the traditional gender stereotypes in the video such as the stereotype that boys are better at math than girls.

However, the negative effect on boys' social utility can hardly be explained by stereotype lift effects. Here, the specific portrait of the boy presented in the stereotyped clip could have played a role. Although the male classmate from whom the girls copied their homework seemed to be mathematically competent, he was also presented as geeky. To the best of our knowledge, effects of this stereotype have not yet been investigated. However, there is research on the stereotypes of math and science. Such research has indicated that favoring these subjects reduces students' perceived social competence and popularity. A study by Hannover and Kessels (2004) showed that students who admitted to liking science were judged as less popular, less attractive, less socially competent, and less integrated than students who claimed they did not like science. As the social utility scale directly referred to social acceptance, the stereotype of the boy as competent but geeky might thus explain the negative effect of the stereotype on boys' social utility.

### Strength and Limitations

One major strength of this study is its high ecological validity. In our experiment, we used a television program that was broadcast on national television. Although the experiment took place in the school context, which does not exactly represent the setting in which children watch television programs in their everyday lives, the experimental material perfectly reflected what children encounter in real-world situations. Contrary to previous research on stereotypes, we furthermore investigated effects of stereotypes embedded into a more complex situation, where a lot of other information was presented to the children. Our results therefore provide initial insights into effects of stereotypes embedded in a television program on young girls and boys in a naturalistic setting. Nevertheless, further studies should also investigate such effects in other real-life settings, such as the home, where children might watch television programs together with their families and therefore might discuss the content of these programs.

In conducting the experiment, we applied a strong research design to address our research questions. We used a randomized block design, randomizing male and female students within classes to the different conditions. Thereby, we investigated possible effects on girls' and boys' performance as well as on different motivational dispositions and attitudes with the aim of obtaining a comprehensive picture of possible effects of traditional stereotypes in television programs. The sample size was based on a power analysis, and in order to increase the transparency of our research, we preregistered all of our hypotheses as well as the analyses. By doing so, we attempted to counter any arguments that might suggest that the effects of stereotype threat were built on *p-hacking* (Flore and Wicherts, 2015).

To assess possible effects of the stereotypes embedded in the television program, we included several different outcome measures such as scales for measuring all dimensions of the task values, for instance, or scales for assessing students' sense of belonging. The findings thus provide a comprehensive picture of possible effects on different outcomes, although one should keep in mind that the scale to assess students' sense of belonging was adapted from the original study. However, the measures we used were based on an achievement test and a questionnaire, which consisted of self-report measures. Our results thus provide no insights into how individuals might process the information presented in the video. Other assessment tools such as observational outcome measures (e.g., eye tracking) are necessary for investigating such processes.

The specific stereotypes transmitted in the television program also need to be considered when interpreting the results of our study. Whereas previous studies on stereotype threat mostly investigated stereotypes of girls being less able to do math than boys (see e.g., Nguyen and Ryan, 2008), the girls in the video might have only been too bored to do their math homework and the boy is depicted as being geeky. The effects on stereotype endorsement indicate that the children noticed the stereotype of boys being better in math than girls in the video. Nonetheless, it is still an open question if a video that more explicitly presents girls as being less able to do math than boys and boys not as being geeky would have caused effects on the other outcomes under investigation. For example, there is research indicating that favoring math and sciences reduces students' perceived social competence and popularity (Hannover and Kessels, 2004). Based on such findings, it can be speculated that the negative effect on social utility for boys found in the present study might be due to the presentation of the boy as being geeky in the video because the social utility scale directly referred to social acceptance. Additionally, it might be possible that the stereotype of the geeky math boy prevented girls from being negatively affected by the video because girls might have experienced this presentation as a negative stereotype against boys. However, such assumptions are rather speculative and further research is necessary to investigate whether other presentations of gender stereotypes affect girls and boys differently than those used in the present study.

Another limitation refers to the sample, which consisted of academic track students (students attending Gymnasiums). We used this sample because academic track schools are the most frequented type of school in Germany (more than 40% of students attend this type of school after primary school), and the school-leaving certificate from academic track schools entitles students to attend university (State Statistical Office of Baden-Württemberg., 2016). When investigating the influence of stereotypes on gender differences in important predictors of STEM careers, it is therefore most informative to assess samples of academic track students. Nevertheless, further research is required to investigate how the results can be generalized to students from other types of schools.

## Conclusion

This study suggests that stereotypes in television can increase children's stereotype endorsement, but hardly affect their motivational dispositions, attitudes, and performance. Consequently, one could argue that traditional gender stereotypes presented in a television programs do not seem to affect young girls in math. This might be positive, particularly in light of the huge amount of time children spend watching television every day (Rideout et al., 2010; Rideout, 2015). However, in our study, we investigated effects of stereotypes in a television program in which only about 1 min of the material had been manipulated, and it might be repeated experience that causes effects to accumulate and sustainably affect boys and girls in the end (Wigfield and Eccles, 2000; Eccles, 2009). Additionally, even such a short clip containing stereotypes presented only once increased children's stereotype endorsement (at least in the short term). The results therefore suggest that television can activate and increase stereotypes about males' advantage in math in children, which might ultimately lead to gender differences in mathematically-intensive STEM fields (Eccles, 2009). Even though we did not find effects on children's motivational dispositions and attitudes, program developers might therefore nonetheless wish to carefully consider including stereotypes in television programs for children.

Our research adds to the discussion of the relevance of stereotype threat effects, particularly with respect to motivational dispositions (see Spencer et al., 2016). Despite effects of the experimental condition on girls' and boys' stereotype endorsement, we found hardly any effects on children's performance, motivational dispositions, and attitudes. Again, it might be repeated experience that renders effects of stereotype threat potentially harmful, and more research is needed to explore the duration of possible effects. Nevertheless, given failed attempts to replicate the original findings on stereotype threat (Stoet and Geary, 2012) and indications of publication bias in the literature on stereotype threat (Flore and Wicherts, 2015), the findings from the present study cast doubt on the robustness of stereotype threat effects. To continue this discussion, it is imperative that non-significant findings are not hidden away in the file drawer.

## Data Availability Statement

Datasets are available on request: The raw data supporting the conclusions of this manuscript will be made available by the authors, without undue reservation, to any qualified researcher.

## Ethics Statement

This study was carried out in accordance with the recommendations of American Psychological Association with written informed consent from all subjects. All subjects and their parents gave written informed consent in accordance with the Declaration of Helsinki. The protocol was approved by the Ethics Committee for Psychological Research of the University of Tübingen.

## Author Contributions

All authors listed have made a substantial, direct and intellectual contribution to the work, and approved it for publication.

### Conflict of Interest Statement

The authors declare that the research was conducted in the absence of any commercial or financial relationships that could be construed as a potential conflict of interest.
