# Supplementary material for: Gender Stereotypes in a Children's Television Program: Effects on Girls' and Boys' Stereotype Endorsement, Math Performance, Motivational Dispositions, and Attitudes
Source: Front Psychol. 2018 Dec 4;9:2435. doi: 10.3389/fpsyg.2018.02435 (PMC6288401; doi:10.3389/fpsyg.2018.02435)
Supplement: Supplementary file 1 [file Table_1.pdf]

Supplementary Material

Gender Stereotypes in a Children's Television Program: Effects on Girls' and Boys' Stereotype Endorsement, Math Performance, Motivational Dispositions, and Attitudes

Eike Wille*, Hanna Gaspard, Ulrich Trautwein, Kerstin Oschatz, Katharina Scheiter, Benjamin Nagengast

*** Correspondence:** Dr. Eike Wille: eike.wille@uni-tuebingen.de

1 Supplementary Tables

1.1 Robustness Check Material (Tables S1-S3)

Because the television program was broadcast on a national TV channel in Germany, we assessed whether participants had already seen the video beforehand, which was the case for 41 students. As a robustness check, we also computed all analyses without these students.

1.2 Sense of Belonging – Full Scale (Table S4 – S6)

Due to low item-scale correlations, we excluded one item when we computed the scale used in the manuscript. Because we did not preregister the exclusion of the item, we conducted the analysis for this outcome also using the original scale, which included all 10 items.

1.3 Robustness Check Without Students Who Did Not Correctly Answer a Question About What They Had Seen in the Last Minute of the Video (Tables S7-S9)

In our analyses, we conducted an intention-to-treat analysis by taking only the original assignment into account in order to keep the randomization to the experimental and control conditions intact. As a robustness check, we ran all analyses without the students who did not correctly answer a question about what they had seen in the last minute of the video, that is, two girls who copied the homework of a classmate in the experimental condition or a summary of the video in the control condition (n = 13). This question was assessed at the end of the posttest questionnaire. The results did not differ meaningfully but are presented in the Supplemental Material.

1.4 Effects on Motivational Dispositions and Attitudes in German (Tables S10-S17)

We preregistered exploratory analyses with respect to motivational dispositions and attitudes in the domain of German. Due to space limitations, the results on girls' and boys' motivation in German are reported in the Supplemental Material.

Table S1

Robustness Check Material 1: Results of Multiple Regression Models for Effects on Stereotype Endorsement, Performance, Self-concept, Sense of Belonging, and Feeling Thermometer

	Stereotype endorsement		Performance		Self-concept		Sense of belonging		Feeling thermometer	
Predictor	β	β 95% CI	β	β 95% CI	β	β 95% CI	β	β 95% CI	β	β 95% CI
Pretest	0.44***	[0.34, 0.54]	0 .86***	[0.81, 0.92]	0.81***	[0.72, 0.89]	0.81***	[0.75, 0.87]	0.85***	[0.78, 0.92]
Gender (boys = 1)	0.26†	[0.01, 0.52]	0.09	[-0.05, 0.23]	-0.02	[-0.15, 0.11]	-0.18*	[-0.33, -0.03]	0.10	[-0.05, 0.24]
Condition (experimental = 1)	0.45**	[0.20, 0.69]	-0.01	[-0.16, 0.15]	0.02	[-0.12, 0.16]	-0.08	[-0.22, 0.06]	-0.11	[-0.24, 0.03]
Gender × Condition	-0.21	[-0.54, 0.11]	-0.02	[-0.19, 0.15]	0.09	[-0.10, 0.28]	0.26*	[0.07, 0.44]	0.05	[-0.14, 0.24]
Effect of condition for boys	0.23†	[0.04, 0.43]	-0.03	[-0.14, 0.09]	0.11	[-0.06, 0.29]	0.18†	[0.02, 0.34]	-0.06	[-0.18, 0.06]

Note. All continuous variables are standardized. Students who stated that they had already seen the video beforehand were excluded (N = 41). The sample consisted therefore of N = 294 students. CI = confidence interval.
† p < .10. * p < .05. ** p < .01. *** p < .001.

Table S2

Robustness Check Material 2: Results of Multiple Regression Models for Effects on Intrinsic Value, Attainment Value, Utility Value for Daily Life, and Cost

	Intrinsic value		Attainment value		Utility value: daily life		Cost	
Predictor	β	β 95% CI	β	β 95% CI	β	β 95% CI	β	β 95% CI
Pretest	0.86 ***	[0.79, 0.94]	0.72 ***	[0.63, 0.81]	0.63 ***	[0.53, 0.73]	0.70 ***	[0.60, 0.80]
Gender (boys = 1)	-0.02	[-0.17, 0.12]	-0.08	[-0.22, 0.07]	-0.08	[-0.32, 0.15]	-0.11	[-0.37, 0.15]
Condition (experimental = 1)	0.03	[-0.13, 0.18]	-0.04	[-0.19, 0.10]	-0.11	[-0.31, 0.09]	-0.05	[-0.22, 0.13]
Gender × Condition	0.01	[-0.20, 0.21]	-0.01	[-0.26, 0.24]	0.03	[-0.25, 0.31]	0.03	[-0.24, 0.31]
Effect of condition for boys	0.03	[-0.12, 0.19]	-0.05	[-0.22, 0.12]	-0.08	[-0.27, 0.11]	-0.01	[-0.28, 0.25]

Note. All continuous variables are standardized. Students who stated that they had already seen the video beforehand were excluded (*N* = 41). The sample consisted therefore of *N* = 294 students. CI = confidence interval.
† $p < .10$. * $p < .05$. ** $p < .01$. *** $p < .001$.

Table S3

Robustness Check Material 3: Results of Multiple Group Multiple Regression Models for Effects on Social Utility Value

	Utility value – social			
	Questionnaire first		Achievement test first	
Predictor	β	β 95% CI	β	β 95% CI
Pretest	0.62 ***	[0.56, 0.69]	0.79 ***	[0.72, 0.87]
Gender (boys = 1)	0.31 **	[0.15, 0.48]	0.10	[-0.24, 0.26]
Condition (experimental = 1)	0.34 †	[0.00, 0.67]	0.16	[-0.01, 0.33]
Gender × Condition	-0.98 ***	[-1.20, -0.76]	-0.03	[-0.37, 0.31]
Effect of condition for boys	-0.64 **	[-0.98, -0.3]	0.13	[-0.08, 0.34]

Note. All continuous variables are standardized. Students who stated that they had already seen the video beforehand were excluded (N = 41). The sample consisted therefore of N = 294 students. CI = confidence interval.
† p < .10. * p < .05. ** p < .01. *** p < .001.

Table S4

Sense of Belonging – Item wording

**German wording**	**English translation**
Im Mathematikunterricht fühle ich mich als wichtiger Bestandteil der Klasse.	I feel like a part of my math class.
Es ist schwer für jemanden wie mich im Mathematikunterricht akzeptiert zu werden (reversed).	It is hard for people like me to be accepted in my math class (reversed).
Andere Schülerinnen und Schüler nehmen mich im Mathematikunterricht ernst.	Other students take my opinions seriously in my math class.
Mein Mathematiklehrer / meine Mathematiklehrerin interessiert sich für mich.	My math teacher is interested in me.
Im Mathematikunterricht fühle ich mich manchmal so, also ob ich nicht dazu gehöre (reversed).	In my math class, sometimes I feel if I don't belong here (reversed).
Im Mathematikunterricht mögen mich meine Mitschülerinnen und Mitschüler so wie ich bin.	Other students in my math class like me the way I am.
Mein Mathematiklehrer / meine Mathematiklehrerin respektiert mich.	My math teacher respects me.
Die anderen wissen, dass ich im Mathematikunterricht gute Arbeit leisten kann.	People in my math class know I can do good work.
Ich wünschte, ich hätte einen anderen Mathematikunterricht (reversed).	I wish I would have a different math class (reversed).
Im Mathematikunterricht fühle ich mich anders als die meisten Schüler und Schülerinnen meiner Klasse (reversed).	I feel very different from most other students in my math class (reversed).

Table S5

Sense of Belonging – Full Scale: Results of Multiple Regression Models

	Sense of belonging	
Predictor	β	β 95% CI
Pretest	0 .81 ***	[0.75, 0.87]
Gender (boys = 1)	-0.19 †	[-0.36, -0.02]
Condition (EG = 1)	-.010	[-0.22, 0.02]
Gender × Condition	0.31 **	[0.12, 0.51]
Effect of condition for boys	0.21 *	[0.04, 0.39]

Note. All continuous variables are standardized. The scale for sense of belonging consisted of ten items, but because of low item-scale correlations (r_{it} = .03/.16), we excluded on item in the computation of the scale used for the analysis in the manuscript. Here, we present results for the original scale, which included all ten items.
† *p* < .10. * *p* < .05. ** *p* < .01. *** *p* < .001.

Table S6

Model Fit for the Confirmatory Factor Analyses

Number of Items	AIC	BIC	CFI	TLI	RMSEA	SRMR
			T1			
10	6785.46	6944.77	0.89	0.78	0.09	0.05
9	6032.10	6168.64	0.92	0.84	0.09	0.05
			T2			
10	6261.43	6418.23	0.95	0.91	0.08	0.05
9	5558.33	5692.73	0.96	0.91	0.08	0.05

Table S7

Robustness Check Without Students Who Did Not Correctly Answer a Question About What They Had Seen in the Last Minute of the Video 1: Results of Multiple Regression Models for Effects on Stereotype Endorsement, Performance, Self-concept, Sense of Belonging, and Feeling Thermometer

	Stereotype endorsement		Performance		Self-concept		Sense of belonging		Feeling thermometer	
Predictor	β	β 95% CI	β	β 95% CI	β	β 95% CI	β	β 95% CI	β	β 95% CI
Pretest	0.36***	[0.22, 0.50]	0.86***	[0.81, 0.92]	0.80***	[0.72, 0.88]	0 .80***	[0.74, 0.86]	0.85***	[0.79, 0.92]
Gender (boys = 1)	0.28†	[0.02, 0.54]	0.09	[-0.07, 0.25]	-0.01	[-0.13, 0.12]	-0.20*	[-0.36, -0.03]	0.04	[-0.10, 0.18]
Condition (experimental = 1)	0.56***	[0.32, 0.80]	0.03	[-0.12, 0.18]	0.05	[-0.07, 0.18]	-0.09	[-0.21, 0.03]	-0.12†	[-0.23, -0.01]
Gender × Condition	-0.33†	[-0.62, -0.03]	-0.05	[-0.22, 0.12]	0.09	[-0.05, 0.24]	0.26*	[0.06, 0.47]	0.10	[-0.08, 0.28]
Effect of condition for boys	0.23*	[0.04, 0.43]	-0.02	[-0.15, 0.10]	0.15	[-0.01, 0.30]	0.18†	[0.01, 0.34]	-0.02	[-0.14, 0.11]

Note. All continuous variables are standardized. Students who failed the manipulation check were excluded (N = 13). The sample consistent therefore of N = 322 students. CI = confidence interval.
† p < .10. * p < .05. ** p < .01. *** p < .001.

Table S8

Robustness Check Without Students Who Did Not Correctly Answer a Question About What They Had Seen in the Last Minute of the Video 2: Results of Multiple Regression Models for Effects on Intrinsic Value, Attainment Value, Utility Value for Daily Life, and Cost

	Intrinsic value		Attainment value		Utility value: daily life		Cost	
Predictor	β	β 95% CI	β	β 95% CI	β	β 95% CI	β	β 95% CI
Pretest	0.86 ***	[0.79, 0.93]	0.71 ***	[0.62, 0.79]	0.63 ***	[0.53, 0.72]	0.71 ***	[0.62, 0.79]
Gender (boys = 1)	-0.03	[-0.18, 0.11]	-0.03	[-0.17, 0.10]	-0.09	[-0.33, 0.14]	-0.14	[-0.37, 0.09]
Condition (experimental = 1)	0.03	[-0.11, 0.17]	-0.02	[-0.16, 0.11]	-0.01	[-0.20, 0.19]	-0.10	[-0.24, 0.05]
Gender × Condition	0.02	[-0.17, 0.20]	0.00	[-0.21, 0.22]	0.00	[-0.29, 0.29]	0.12	[-0.11, 0.34]
Effect of condition for boys	0.05	[-0.10, 0.19]	-0.02	[0.00, 0.00]	-0.02	[-0.18, 0.14]	0.02	[-0.21, 0.25]

Note. All continuous variables are standardized. Students who failed the manipulation check were excluded (*N* = 13). The sample consistent therefore of *N* = 322 students. CI = confidence interval.
† $p < .10$. * $p < .05$. ** $p < .01$. *** $p < .001$.

Table S9

Robustness Check Without Students Who Did Not Correctly Answer a Question About What They Had Seen in the Last Minute of the Video 3: Results of Multiple Group Multiple Regression Models for Effects on Social Utility Value

	Utility value – social			
	Questionnaire first		Achievement test first	
Predictor	β	β 95% CI	β	β 95% CI
Pretest	0.66 ***	[0.60, 0.73]	0.75 ***	[0.69, 0.82]
Gender (boys = 1)	0.30 **	[0.14, 0.47]	0.13	[-0.11, 0.37]
Condition (experimental = 1)	0.24	[-0.01, 0.49]	0.24 *	[0.07, 0.42]
Gender × Condition	-0.88 ***	[-1.07, -0.70]	-0.11	[-0.42, 0.21]
Effect of condition for boys	-0.65 ***	[-0.93, -0.37]	0.14	[-0.05, 0.33]

Note. All continuous variables are standardized. Students who failed the manipulation check were excluded (*N* = 13). The sample consistent therefore of *N* = 322 students. CI = confidence interval.
† $p < .10$. * $p < .05$. ** $p < .01$. *** $p < .001$.

Table S10

Effects on Motivation in German 1: Descriptive Statistics for All Study Variables on the Pretest Separated by Gender

	Girls		Boys				
Variable	*M*	*SD*	*M*	*SD*	*d*[a]	*d* 95% CI	
Self-concept T1	3.07	0.08	2.83	0.08	-0.32	-0.50	-0.14
Sense of belonging T1	3.14	0.06	2.30	0.05	-0.27	-0.49	-0.05
Intrinsic value T1	3.05	0.11	2.65	0.08	-0.48	-0.69	-0.23
Attainment value T1	3.57	0.07	3.33	0.05	-0.38	-0.64	-0.12
Utility value – daily life T1	3.09	0.08	2.93	0.09	-0.21	-0.43	0.00
Utility value – social T1	2.05	0.05	1.99	0.05	-0.09	-0.02	0.06
Cost T1	1.56	0.06	1.80	0.07	0.36	0.18	0.54

Note. CI = 95% confidence interval.
[a]The dependent variable is standardized.

Table S11

Effects on Motivation in German 3: Descriptive Statistics for All Study Variables at T1 Separated by Gender and Group

	Girls								Boys							
	Experimental group				Control group				Experimental group				Control group			
Variable	*M*	*SD*	Min	Max	*M*	*SD*	Min	Max	*M*	*SD*	Min	Max	*M*	*SD*	Min	Max
Self-concept T1	3.07	0.08	1.00	4.00	3.07	0.11	1.00	4.00	2.77	0.08	1.00	4.00	2.88	0.11	1.00	4.00
Sense of belonging T1	3.11	0.07	2.11	4.00	3.18	0.08	1.78	4.00	2.93	0.06	1.44	3.89	3.07	0.07	2.11	4.00
Intrinsic value T1	3.04	0.12	1.00	4.00	3.07	0.14	1.00	4.00	2.63	0.10	1.00	4.00	2.68	0.13	1.00	4.00
Attainment value T1	3.61	0.08	1.25	4.00	3.52	0.09	1.25	4.00	3.32	0.06	1.75	4.00	3.35	0.09	1.75	4.00
Utility Value – daily life T1	3.14	0.09	1.00	4.00	3.02	0.10	1.33	4.00	2.78	0.09	1.33	4.00	3.07	0.12	1.00	4.00
Utility value – social T1	2.07	0.08	1.00	3.67	2.03	0.10	1.00	4.00	1.92	0.08	1.00	3.67	2.07	0.07	1.00	3.67
Cost T1	1.61	0.07	1.00	3.50	1.50	0.09	1.00	3.25	1.83	0.08	1.00	3.75	1.76	0.11	1.00	3.50

Table S12

Effects on Motivation in German 4: Descriptive Statistics for All Study Variables at T2 Separated by Gender and Group

	Girls								Boys							
	Experimental group				Control group				Experimental group				Control group			
Variable	*M*	*SD*	Min	Max	*M*	*SD*	Min	Max	*M*	*SD*	Min	Max	*M*	*SD*	Min	Max
Self-concept T2	3.00	0.07	1.25	4.00	3.08	0.08	1.00	4.00	2.86	0.08	1.00	4.00	2.94	0.09	1.00	4.00
Sense of belonging T2	3.10	0.05	2.00	4.00	3.08	0.06	1.78	4.00	3.06	0.05	1.44	4.00	3.01	0.07	1.33	4.00
Intrinsic value T2	2.89	0.10	1.25	4.00	2.84	0.09	1.00	4.00	2.81	0.09	1.00	4.00	2.78	0.12	1.00	4.00
Attainment value T2	3.48	0.05	1.50	4.00	3.49	0.05	1.25	4.00	3.43	0.07	1.75	4.00	3.37	0.10	1.00	4.00
Utility value – daily life T2	3.06	0.09	1.00	4.00	3.05	0.07	1.67	4.00	2.89	0.07	1.00	4.00	3.02	0.10	1.00	4.00
Utility value – social T2	2.12	0.06	1.00	4.00	1.90	0.05	1.00	4.00	2.01	0.07	1.00	3.67	2.10	0.07	1.00	3.67
Cost T2	1.58	0.06	1.00	3.25	1.66	0.08	1.00	3.75	1.66	0.07	1.00	4.00	1.79	0.08	1.00	4.00

Table S13

Effects on Motivation in German 5: Correlations Between all Study Variables in German

	Variable	1.	2.	3.	4.	5.	6.	7.	8.	9.	10.	11.	12.	13.	14.
1.	Self-concept T1	—													
2.	Self-concept T2	.86	—												
3.	Sense of belonging T1	.64	.58	—											
4.	Sense of belonging T2	.61	.66	.78	—										
5.	Intrinsic value T1	.72	.69	.59	.52	—									
6.	Intrinsic value T2	.73	.78	.56	.63	.86	—								
7.	Attainment value T1	.35	.37	.30	.30	.45	.45	—							
8.	Attainment value T2	.32	.35	.26	.38	.37	.44	.76	—						
9.	Utility value – daily life T1	.28	.25	.33	.25	.46	.40	.33	.29	—					
10.	Utility value – daily life T2	.35	.39	.31	.35	.45	.48	.36	.35	.75	—				
11.	Utility value – social T1	.19	.18	.21	.17	.30	.25	.28	.21	.28	.30	—			
12.	Utility value – social T2	.21	.17	.16	.19	.18	.22	.24	.21	.19	.25	.66	—		
13.	Cost T1	-.73	-.69	-.56	-.52	-.69	-.68	-.31	-.28	-.34	-.39	-.12	-.07	—	
14.	Cost T2	-.64	-.73	-.49	-.63	-.58	-.68	-.27	-.36	-.26	-.37	(-.09)	(-.03)	.78	—

Note. Non-significant correlations are displayed in parentheses, for all other correlations, $p < .05$.

Table S14

Effects on Motivation in German 6: Results of Multiple Regression Models for Outcomes in German

	Intrinsic value			Utility - daily life		
Predictor	β		β 95% CI	β		β 95% CI
Pretest	0.84	***	[0.79, 0.90]	0.73	***	[0.68, 0.79]
Gender (boys = 1)	-0.07		[-0.27, 0.13]	-0.04		[-0.25, 0.17]
Condition (experimental = 1)	0.06		[-0.08, 0.21]	0.02		[-0.16, 0.20]
Gender × Condition	-0.03		[-0.23, 0.17]	-0.17		[-0.44, 0.09]
Effect of condition for boys	0.03		[-0.20, 0.41]	-0.16		[-0.46, 0.22]

Note. All continuous variables are standardized. CI = confidence interval.
† *p* < .10. * *p* < .05. ** *p* < .01. *** *p* < .001.

Table S15

Effects on Motivation in German 7: Results of Multiple Group Multiple Regression Models for Effects on Self-concept and Sense of Belonging in German

	Self-concept				Sense of belonging			
	Questionnaire first		Achievement test first		Questionnaire first		Achievement test first	
Predictor	β	β 95% CI	β	β 95% CI	β	β 95% CI	β	β 95% CI
Pretest	0.84 ***	[0.75, 0.93]	0.85 ***	[0.83, 0.88]	0.73 ***	[0.62, 0.84]	0.81 ***	[0.76, 0.87]
Gender (boys = 1)	-0.05	[-0.26, 0.16]	-0.30 *	[-0.51, -0.08]	-0.05	[-0.32, 0.23]	-0.21 †	[-0.42, 0.00]
Condition (experimental = 1)	0.10	[-0.02, 0.21]	-0.30 ***	[-0.46, -0.15]	0.21	[-0.03, 0.44]	-0.12	[-0.27, 0.03]
Gender × Condition	-0.15	[-0.33, 0.03]	0.17	[-0.07, 0.41]	-0.19	[-0.46, 0.08]	0.25 *	[0.06, 0.44]
Effect of condition for boys	-0.06	[-0.35, 0.35]	-0.13	[-0.13, 0.47]	0.01	[-0.46, 0.58]	0.13	[-0.02, 0.71]

Note. All continuous variables are standardized. CI = confidence interval.
† p < .10. * p < .05. ** p < .01. *** p < .001.

Table S16

Effects on Motivation in German 8: Results of Multiple Group Multiple Regression Models for Effects on Attainment Value and Social Utility Value in German

	Attainment value				Social utility value			
	Questionnaire first		Achievement test first		Questionnaire first		Achievement test first	
Predictor	β	β 95% CI	β	β 95% CI	β	β 95% CI	β	β 95% CI
Pretest	0.76***	[0.68, 0.85]	0.75***	[0.61, 0.89]	0.69***	[0.64, 0.74]	0.66***	[0.58, 0.74]
Gender (boys = 1)	0.10	[-0.12, 0.32]	-0.44†	[-0.86, -0.02]	0.30*	[0.08, 0.52]	0.27†	[0.03, 0.51]
Condition (experimental = 1)	0.15	[-0.04, 0.34]	-0.17†	[-0.33, -0.01]	0.13	[-0.08, 0.33]	0.50***	[0.28, 0.73]
Gender × Condition	-0.33	[-0.71, 0.05]	0.48*	[0.08, 0.88]	-0.38†	[-0.7, -0.06]	-0.53*	[-0.9, -0.17]
Effect of condition for boys	-0.18	[-0.78, 0.23]	0.31	[0.05, 1.45]	-0.25†	[-0.98, -0.13]	-0.03	[-0.71, 0.10]

Note. All continuous variables are standardized. CI = confidence interval.
† $p < .10$. * $p < .05$. ** $p < .01$. *** $p < .001$.

Table S17

Effects on Motivation in German 9: Results of Multiple Group Multiple Regression Models for Effects on Cost in German

	Cost			
	Questionnaire first		Achievement test first	
Predictor	β	β 95% CI	β	β 95% CI
Pretest	0.75***	[0.63, 0.86]	0.78***	[0.70, 0.87]
Gender (boys = 1)	0.05	[-0.15, 0.24]	0.32†	[0.04, 0.61]
Condition (experimental = 1)	-0.31**	[-0.48, -0.14]	0.09	[-0.15, 0.34]
Gender × Condition	0.05	[-0.31, 0.40]	-0.21	[-0.51, 0.10]
Effect of condition for boys	-0.26	[-0.83, 0.22]	-0.11	[-0.85, -0.02]

Note. All continuous variables are standardized. CI = confidence interval.
† p < .10. * p < .05. ** p < .01. *** p < .001.
